# Micro-Organisms as a Cause of Chronic Disease

**Published:** 1900-02-03

**Authors:** 


### HOSPITAL CLINICS AND MEDICAL PROGRESS.

### MICRO-ORGANISMS AS A CAUSE OF CHRONIC DISEASE.

THE science of bacteriology has become so precise, and the methods by which the more direct relations between microbes and disease can be traced out are so well understood, that imagination is at a discount, and, properly enough, the man who propounds hypotheses is straightway asked for proofs. Still, when one deserts the beaten track a little speculation is allowable, and thus it may be permissible to inquire, even though no definite answer be at present possible, whether microbes are not concerned in the production of many diseases of a chronic character which have hitherto been attributed to more ordinary causes, or have even been distinguished by that most unsatisfactory appellation "idiopathic." We know that microbes when they gain a footing in the body may set up fevers and diseases of various kinds. We know also that when established locally they may set up a local reaction, taking the form of an inflammation or overgrowth of tissue, and that by their continued multiplication and the reaction it sets up this new formation may spread widely, as in the case of tuberculosis. The question, however, which is now in the air is, What is the effect upon the body of being constantly worried by microbes whose attack is so feeble that they never do gain a footing, but are always beaten off? Does the body under such circumstances, although never infected by these microbes in the true sense of the word, yet suffer injury from their presence? It must be admitted that the observations so far to hand on this point are somewhat inconclusive, and that other explanations may perhaps be forthcoming; but, as things stand, the drift of recent investigations points to the possibility that microbes of one kind and another gain access to the various organs and even to the blood far more constantly than many people imagine, and that, although such microbes may be ultimately destroyed, so that none of the symptoms which characterise an overmastering infection may be produced, still the prolonged contest between the greater organisms and its multitudinous assailants is not without effect. Victory is bought at a certain cost, the traces of the fight showing themselves as chronic disease of various kinds. As an example we may take cirrhosis of the liver. What has ever been more widely accepted as one of the verities of our faith than that the gin-drinker's liver is the result of drinking ardent spirits? Yet who has not known some of the jolliest of topers die without a mark of the hobnail, and who has not heard of cases where the dead-house has told a secret which, if true, had certainly been well kept during life? There must be something else—shall we call it some accidental circumstance, besides the alcohol, to cause the one patient to be affected and the other not? Is that accidental circumstance a microbe?

Let us consider the matter in the light shed upon it by the researches of Professor Adami, of Montreal, as described in an address recently delivered before the Society of Internal Medicine at Chicago. What Professor Adami shows is that although the various organs of the healthy body are practically free from micro-organisms, they are not absolutely so, and that such comparative sterility as they enjoy in normal health is not due to these micro-organisms being unable to get in, but to the constant activity of endothelial and other cells, by which they are destroyed within the body. We do not walk like knights of old, armed cap-à-pie, so that none of the common herd of enemies with which we are surrounded can gain access through our impervious mucous membranes. On the contrary, microbes of all sorts—good, bad, and indifferent—are constantly getting in. Our defence lies in the vitality of the cells by which these microbes are digested and destroyed. If these fail then we may have some general disorder. But, without failing, the cells engaged in this depurating process may be so hard pressed and overworked that they themselves may become diseased, and according to the particular organ on which the stress has fallen local maladies may be set up. Such is the theory.

To turn back again, then, to the particular disease we were considering—"hobnail" liver. If the small intestines of the healthy rabbit be taken immediately after death, and sections be cut and stained, abundant leucocytes containing micro-organisms are found in the lymph follicles of the Peyer's patches. The tendency is for the bacteria so introduced to be destroyed within the lymph glands of the submucous tissue; but occasions may arise in which this destruction is inadequate, and then by proper search they may be found in the lymphatics of the mesentery.

But what happens to the lymphatics happens also to the venules of the portal system. Into these venous radicles bacteria are constantly being introduced, and they have to be dealt with by the cells of the liver. Thus in health the body is kept practically, although not absolutely free from microbic infection, not by virtue of its own immunity, or the imperviousness of its mucous membranes, but as the result of work done by certain groups of cells, among which are those upon which the blood flowing up from the intestinal collecting ground first impinges in the liver. But suppose that, as the result of chronic intestinal catarrh, or other cause, an excessive number of bacteria are absorbed by the portal system, then, although these bacteria may still be destroyed or rendered harmless by the action of the liver cells, "the excessive action of these cells, and the effect upon them of the bacterial toxins liberated in the process of destruction, may nevertheless lead to grave changes in these cells and in the organs of which they are a part; changes which are of a chronic nature." Here then is the suggestion as to the origin of cirrhosis: the chronic inflammatory changes, and the atrophy which characterise this disease, are not the direct effects of alcohol, but arise from the stress, the overwork, and the poisoning of the tissues caused by the destruction of so many microbes, these being derived from the catarrhal intestinal mucous membrane constantly found with this disease. If one could be sure that this was the true explanation of the hobnail liver many other diseased conditions might be hung upon the same peg. Hence the interest which attaches to what we fear must yet be called only a speculation. Adami has, however, shown that in cases of hæmochromatosis the fine masses of pigment found are in a large proportion of cases merely modified bacteria. We may have, he says, "a succession of cases in which at first only the abdominal walls and the mesenteric glands become the seat of the deposit of this pigment, and to a slight extent the liver; then cases in which the liver is seriously involved with associated or accompanying cirrhosis; and in still others in which the pancreas also becomes the seat of this abundant deposit in the cells of minute pigmented granules, which are the modified 'corpses' of bacteria"; and he goes on to say that the similar pigmented granules, which are found in the livers in cases of pernicious anæmia, are not the result of disintegration of red blood-corpuscles, but in that disease also are evidences of a condition of subinfection—the chronic inflammatory condition of the upper digestive tract being associated with excessive passing into the portal blood of colon and allied bacilli, which are destroyed in the liver. In regard to this question the researches of Dr. William Hunter[2] in regard to the origin of pernicious anæmia are of very great interest and importance, for he also has been led to look upon this disease as due to a chronic infective process originating in the digestive tract. Thus we seem to have researches in different directions pointing to the conclusion that the contest with an infection may be productive of serious harm to the organism, not merely when the microbes gain a footing, but also when they are destroyed, and that certain chronic diseases are but expressions of the effort which is being or has been put forth by the tissues of the body —the mucous membranes, the blood, or the more solid organs—in beating off its multitudinous assailants.

[1] New York Med. News, Jan. 6. [2] Lancet, Jan. 27.

